# Selective transcriptomic dysregulation of metabolic pathways in liver and retina by short- and long-term dietary hyperglycemia

**DOI:** 10.1016/j.isci.2024.108979

**Published:** 2024-01-19

**Authors:** Anupam K. Mondal, Daniel C. Brock, Sheldon Rowan, Zhi-Hong Yang, Krishna Vamsi Rojulpote, Kelsey M. Smith, Sarah G. Francisco, Eloy Bejarano, Milton A. English, Amy Deik, Sarah Jeanfavre, Clary B. Clish, Alan T. Remaley, Allen Taylor, Anand Swaroop

**Affiliations:** 1 Neurobiology Neurodegeneration & Repair Laboratory, National Eye Institute (NEI), National Institutes of Health (NIH), Bethesda, MD, USA; 2 Laboratory for Nutrition & Vision Research, JM-USDA Human Nutrition Research Center on Aging, Tufts University, Boston, MA, USA; 3 Friedman School of Nutrition Science and Policy, and Department of Molecular and Chemical Biology, Tufts University, Boston, MA, USA; 4 Department of Ophthalmology, Tufts University School of Medicine, Boston, MA, USA; 5 Lipoprotein Metabolism Section, Translational Vascular Medicine Branch, National Heart, Lung and Blood Institute (NHLBI), National Institutes of Health (NIH), Bethesda, MD, USA; 6 Broad Institute of MIT and Harvard, Cambridge, MA, USA; 7 School of Health Sciences and Veterinary School, Universidad CEU Cardenal Herrera, CEU Universities, Valencia, Spain

**Keywords:** Physiology, Metabolomics, Transcriptomics

## Abstract

A high glycemic index (HGI) diet induces hyperglycemia, a risk factor for diseases affecting multiple organ systems. Here, we evaluated tissue-specific adaptations in the liver and retina after feeding HGI diet to mice for 1 or 12 month. In the liver, genes associated with inflammation and fatty acid metabolism were altered within 1 month of HGI diet, whereas 12-month HGI diet-fed group showed dysregulated expression of cytochrome P450 genes and overexpression of lipogenic factors including *Srebf1* and *Elovl5*. In contrast, retinal transcriptome exhibited HGI-related notable alterations in energy metabolism genes only after 12 months. Liver fatty acid profiles in HGI group revealed higher levels of monounsaturated and lower levels of saturated and polyunsaturated fatty acids. Additionally, HGI diet increased blood low-density lipoprotein, and diet-aging interactions affected expression of mitochondrial oxidative phosphorylation genes in the liver and disease-associated genes in retina. Thus, our findings provide new insights into retinal and hepatic adaptive mechanisms to dietary hyperglycemia.

## Highlights

• High glycemic index diet has distinct impact on metabolic genes in the liver and retina
• HGI diet raises hepatic levels of monounsaturated fatty acids and blood cholesterol
• Blood levels of LDL-cholesterol significantly increase after long-term HGI diet
• Diet-aging interaction distinctly alters gene expression in the liver and retina

## Graphical abstract

**Figure:**
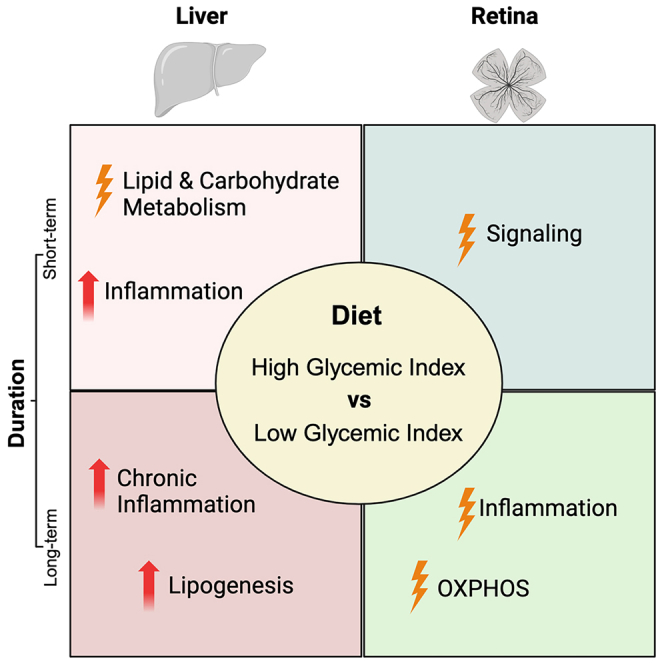

## Introduction

Diet, along with other lifestyle choices, is a major contributing factor to debilitating diseases that affect millions of humans globally.^1^ Numerous studies have uncovered associations between dietary carbohydrate intake with lifespan and mortality.^2^^,^^3^ Dietary glycemic exposure impacts blood-glucose levels, which when unchecked is a major etiologic factor for obesity and diabetes.^4^ High blood-glucose levels, also known as hyperglycemia, result from consumption of high glycemic index (HGI) carbohydrates, which are rapidly broken down into glucose postprandially and associated with rapid spikes and prolonged elevation of blood-glucose levels. Chronic hyperglycemia can lead to higher HbA1c, a measure that integrates glucose exposure over a few months. It also leads to insulin resistance and irregular metabolic control across multiple organ systems, resulting in increased risk for type 2 diabetes, cardiovascular disease, and obesity.^5^^,^^6^ These consequences are compounded by aging, leaving the elderly population particularly vulnerable.^7^ The role of dietary hyperglycemia in healthy aging and the prospect of diet modulation for preventing age-related metabolic dysfunctions are areas of active investigation.^6^^,^^8^ In comparison to HGI, a low glycemic index (LGI) diet improves regulation of blood glucose and is suggested for limiting or reversing certain disease risks.^4^^,^^9^ To encourage wider adoption of healthy dietary choices, it is crucial to characterize the glycemic index (GI) of food items and their molecular impact on human health and aging.^10^

The primary effect of an HGI diet is elevated levels of glucose interfering with normal insulin-regulated metabolism over time. Excess glucose poses unique challenges in different tissues. The liver is the foremost organ exposed to postprandial nutrients and is tasked with regulating glucose homeostasis.^11^ The liver stores extra glucose as glycogen but excessive supply, such as that from HGI diet, can result in conversion to fatty acids via *de novo* lipogenesis leading to liver steatosis and other liver pathologies.^12^ On the other hand, the brain and central nervous systems rely on glucose for energy needs; yet HGI diet is correlated with higher cerebral amyloid burden, age-related macular degeneration (AMD), and depression in humans.^6^^,^^9^^,^^13^^,^^14^^,^^15^^,^^16^ Interestingly, dietary GI responses can vary among individuals,^17^^,^^18^ indicating that additional complexities such as genetic variations, microbiome, environmental factors, and aging might exert tissue-specific adaptive responses to dietary GI.

Given their different functions, location, environment and exposure, we hypothesize that tissues adapt differently to dietary glycemic exposure and that the response is dependent upon age and duration of diet consumption.^19^ To test this hypothesis, we chose to study two distinct metabolically demanding organs—the liver and the retina. Both the liver and the retina have documented hyperglycemia and aging related pathologies. Interesting differences between the two tissues include their potential to proliferate and their distinct metabolic profiles. The liver is remarkable for its ability to regrow following injury, whereas the retina is a post-mitotic neuronal tissue incapable of cellular regeneration.^20^ Liver metabolism is flexible, with hepatocytes utilizing diverse sources of fatty acids and glucose as fuel, dependent on hormones and nutrient availability.^21^ In contrast, the neural retina relies primarily on glucose metabolism and to a much lesser extent beta-oxidation to support phototransduction and neurotransmission.^22^^,^^23^^,^^24^ The metabolic interactions between the liver and retina are complex, spanning multiple pathways involving metabolism, mitochondria, oxidative stress, and inflammation.

Recent technological advances have enabled dissection of complex biological systems at unprecedented resolution, with model organisms being useful for delineating at least some of the molecular effects of dietary glycemic exposure.^19^^,^^25^^,^^26^^,^^27^^,^^28^^,^^29^^,^^30^ High-throughput “omics” studies have revealed the impact of HGI diet on retinal structure and function.^19^^,^^31^ Interestingly, changing from HGI to LGI diet later in life was shown to arrest or even partially reverse some of the changes. Omics studies have also been useful for elucidating adaptive responses and aging-related alterations in various tissues.^32^ Integration of “omics” data from multiple tissues will help delineate the complex biological processes underlying tissue-specific response to individual dietary variables such as dietary glycemic exposure. To test our hypothesis, we fed male C57BL/6J mice isocaloric HGI and LGI diets for one-month (1-mon) and 12-mon duration and performed global transcriptome analyses of the liver and retina. Our analyses have uncovered alterations in hepatic inflammation and lipid metabolic processes after 1-mon of HGI diet. HGI further exacerbates the dysregulated fatty acid metabolism pathways in the liver after 12-mon consumption. Notably, retinal response to HGI and LGI is not prominent after one month, but significant shifts in oxidative phosphorylation and inflammation pathways are evident after 12-mon feeding. Our study therefore provides mechanistic insights into the response patterns to dietary GI in the liver and retina.

## Results

### Study design

**Figure 1:**
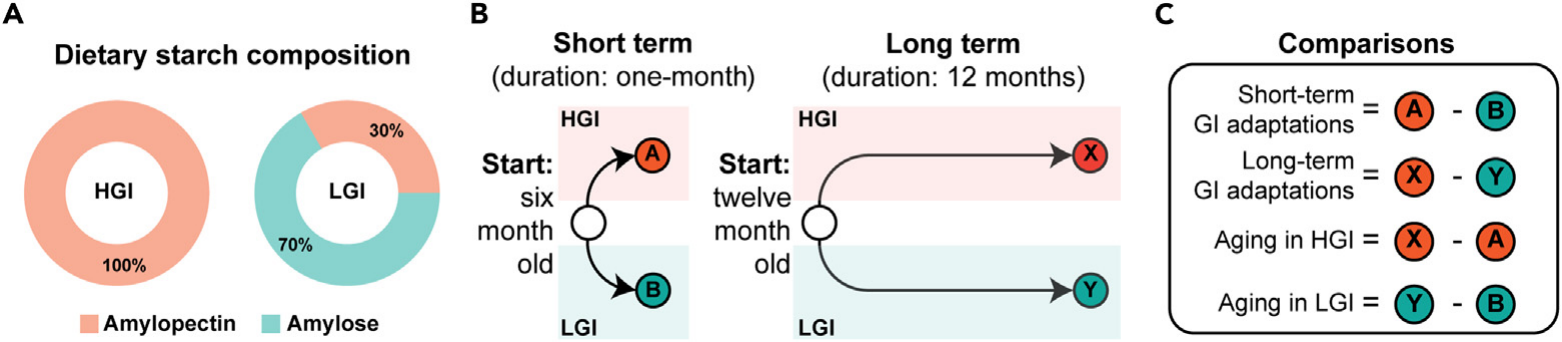
Study design to investigate molecular adaptations to dietary glycemic exposure and aging (A) Male C57BL/6J mice were fed either an HGI diet containing starch composing 100% amylopectin or an LGI diet with starch that had 70% amylose and 30% amylopectin. (B) Short-term treatments were performed in six-month-old mice for a period of 1 month. Long-term treatments were performed in twelve-month-old mice and lasted for a total of 12 months. (C) Comparisons performed to test our hypothesis.

We hypothesized that dietary response and aging intersect to yield unique tissue-specific adaptations. To test our hypothesis, we set up a two-pronged feeding schedule and selected two key tissues—the liver and the retina (Figure 1). To isolate the effects of dietary GI, we designed isocaloric HGI and LGI diets that only varied in starch composition. The HGI diet contained 100% amylopectin, whereas the LGI diet contained a combination of 30% amylopectin and 70% amylose (Figures 1A and Table S1). Amylopectin is rapidly digested, whereas the breakdown of amylose is slower. Both diets provide the same, adequate amounts of micronutrients and are isocaloric. Short-term feeding (one-month, 1-mon) of HGI or LGI diet was investigated in six-month-old mice (Figure 1B). For long-term adaptations, twelve-month-old mice were fed HGI or LGI for twelve months. Our study design allowed us to identify duration-dependent tissue-specific GI responsive pathways and inspect aging-related shifts within each glycemic treatment (Figure 1C).

### One-month HGI feeding impacts gene expression for core liver metabolic pathways and inflammation

**Figure 2:**
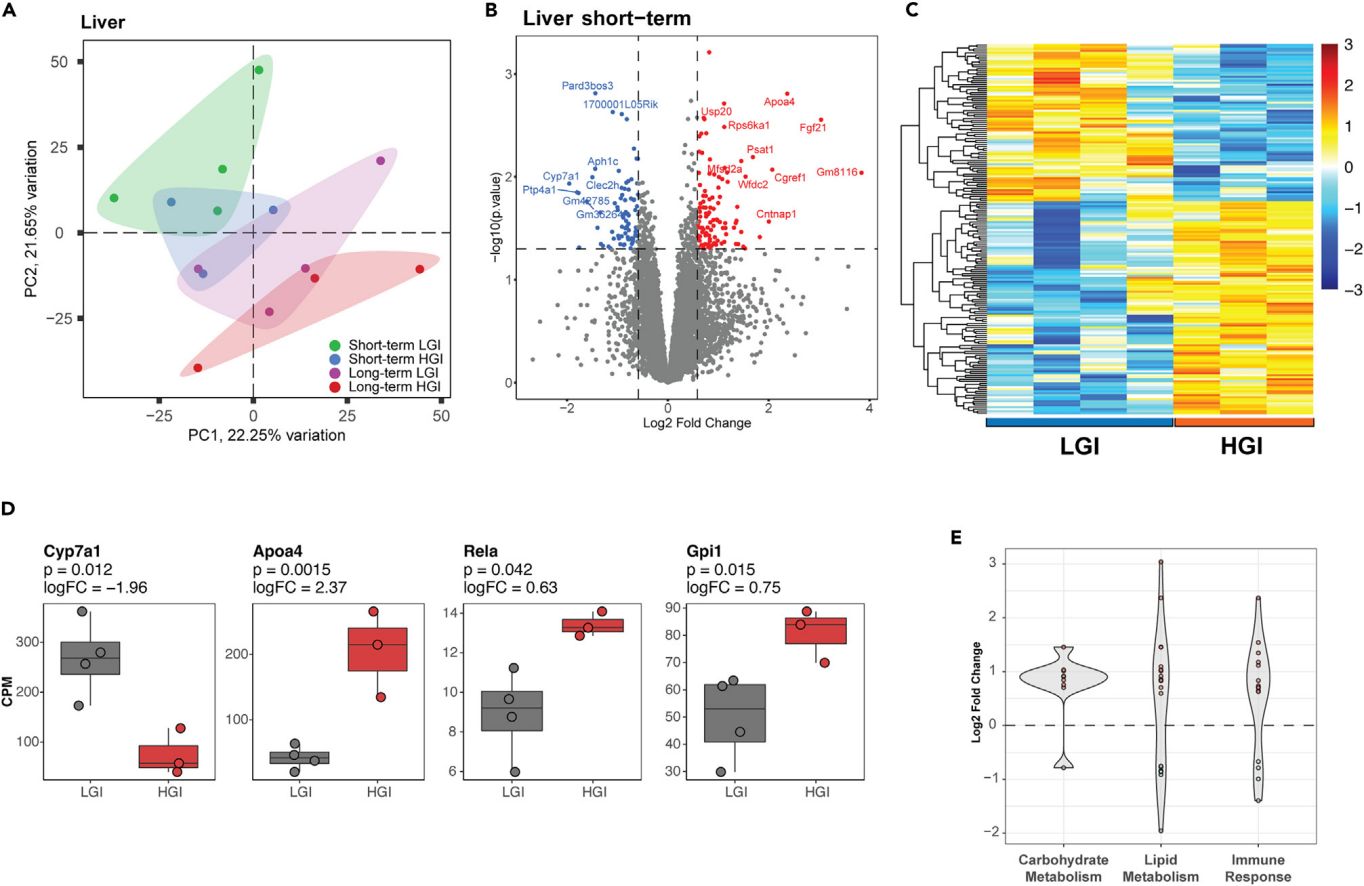
Short-term HGI diet alters hepatic transcriptome (A) Liver transcriptomes cluster by diet and length of feeding in principal-component analysis (PCA). (B) Volcano plot of significant DEGs in livers of short term HGI- versus LGI-fed animals using a p value <0.05 and absolute l log_2_(fold change) ≥ 1.5. (C) Heatmap showing gene expression trends of short-term HGI-linked DEGs in liver. (D) Expression of selected significant DEGs in livers of HGI and LGI animals. See Table S2 for additional details. (E) Short-term HGI diet alters expression of lipid and carbohydrate metabolism- and immune response-related genes. Gene sets were collated from the following gene ontology (mouse genome informatics [MGI]) terms: lipid metabolic processes, carbohydrate metabolism, and immune response.

The RNA sequencing (RNA-seq) experiment captured expression of 11,742 genes in the liver samples, which clustered according to diet and length of feeding in principal-component analysis (PCA) (Figure 2A). Differential expression analysis, as indicated in Figure 1C, revealed significant transcriptomic alterations in livers of HGI- versus LGI-fed animals after one month (Figures 2B and 2C). A total of 186 genes qualified as differentially expressed genes (DEGs) using a p value <0.05 and absolute fold change ≥1.5. Among the top DEGs in HGI vs. LGI livers were *ApoA4* (2.37 log_2_ fold change [logFC]) and *Crat* (1.1 logFC) exhibiting higher expression, and *Cyp7a1* (−1.96 logFC) that functions in interconversion of acetyl-coenzyme A (CoA) and acetylcarnitine and bile acid synthesis showing reduced expression, (Figure 2D and Table S2). Gene set enrichment analysis (GSEA) identified 41 pathways impacted by transcriptomic differences between short-term HGI- and LGI-fed animals (Table S2). Interleukin-3, interleukin-5, and granulocyte-macrophage colony-stimulating factor (GM-CSF) signaling, gluconeogenesis, and triglyceride and fatty acid metabolism were among the enriched gene sets in HGI livers, with cholesterol biosynthesis and respiratory electron transport being negatively enriched.

To obtain a broad picture of diet and hepatic gene expression, we focused on DEGs-enriched pathways that could be grouped into three broader categories of carbohydrate metabolism, lipid and fatty acid metabolism, and inflammation and immune response. Carbohydrate metabolism and gluconeogenesis genes, including glucokinase (*Gck*, 0.91 logFC) and glucose-6-phosphate isomerase 1 (*Gpi1*, 0.75 logFC), exhibited higher expression in the HGI group (Figures 2D and 2E). Interestingly, Gpi1 is shown to have secondary functions in immune response. Enhanced expression of *Gpi1* together with *Rela* (p65 or nuclear factor κB [NF-κB], 0.63 logFC) indicates HGI diet-induced inflammation. Lipid metabolism genes mostly showed higher expression in response to HGI. We also noted reduced expression of the gene set for respiratory electron transport in the HGI group (Table S2). Short-term HGI diet also altered expression of multiple carbohydrate and lipid metabolism-, immune response-, and inflammation-related genes (Figure 2E and Table S2). Thus, transcriptomic analysis demonstrated significant changes in genes associated with metabolic and inflammation pathways in the liver as early as 1-mon of feeding the HGI diet.

### Long-term HGI diet enhances hepatic expression of genes associated with very long chain fatty acids and chronic inflammation

**Figure 3:**
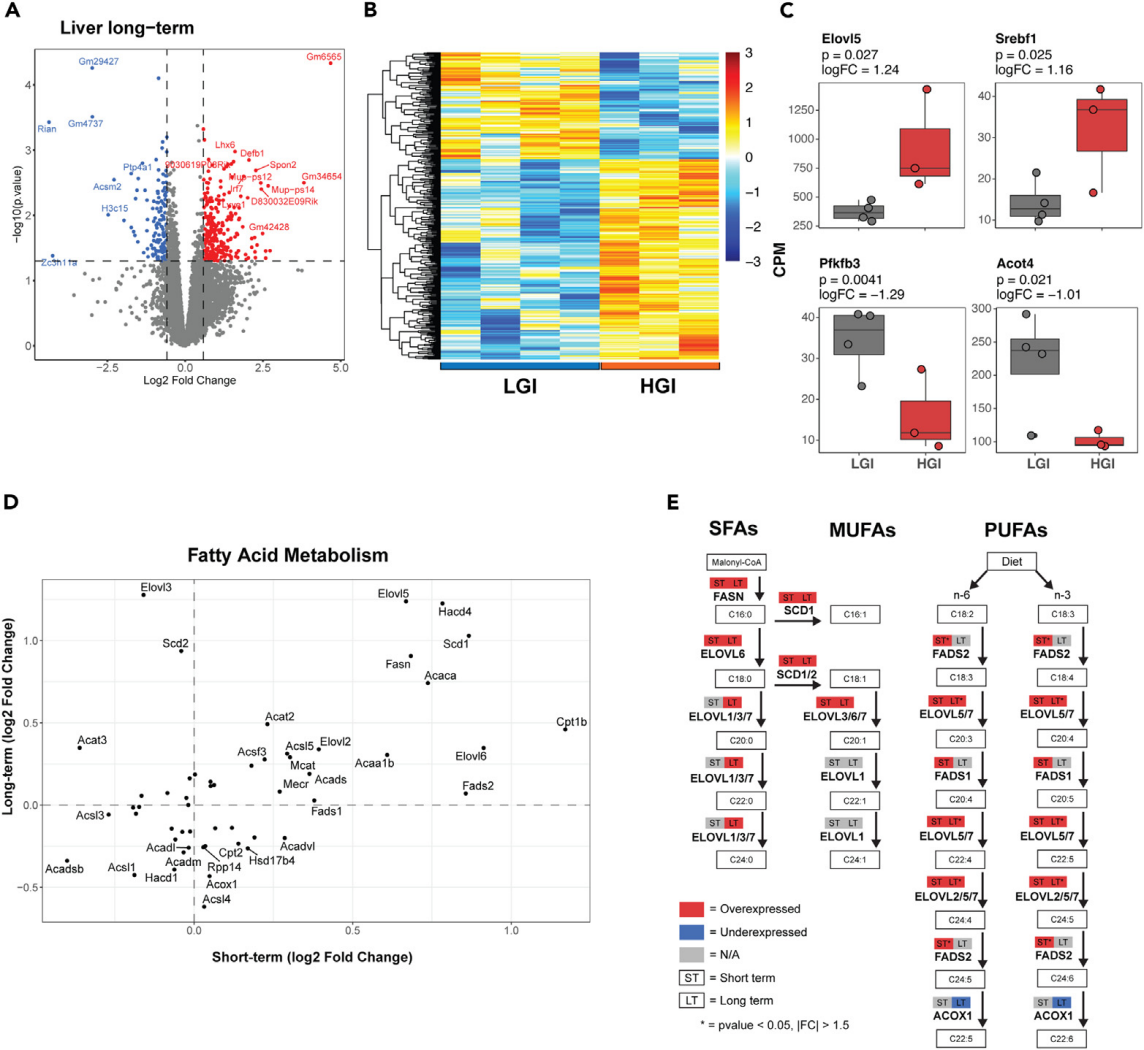
Long-term HGI diet produces significant transcriptomic alterations in key fatty acid metabolic processes of the liver (A) Volcano plot of DEGs in the liver after long-term HGI diet using a p value <0.05 and absolute fold change ≥1.5. (B) Heatmap of DEGs in liver after long-term HGI vs. LGI feeding. (C) Important liver fatty acid and glycolytic enzymes respond to long-term HGI diet. (D) Comparison of transcriptomic outline of fatty acid metabolism pathway in short- versus long-term HGI treatment. Genes were obtained from KEGG (mmu01212). The x axis denotes log_2_ fold change after short-term feeding whereas the y axis represents log_2_ fold change after long-term HGI. (E) HGI-linked transcriptional changes in fatty acid elongation pathway for saturated fatty acids (SFAs), monounsaturated fatty acids (MUFAs), and polyunsaturated fatty acids (PUFAs). Fatty acid species are shown in boxes with gene names shown in bold text. Boxes above gene names represent over expression (positive logFC, shown in red), under expression (negative logFC, shown in blue), or no change shown in gray in short-term and long-term HGI diets. ∗ indicates statistical significance with a p value <0.05.

Transcriptomic comparison of HGI versus LGI diet fed for 12-mon resulted in significantly higher expression of 259 and lower expression of 138 genes in livers of HGI-fed mice (p value <0.05 and fold change ≥1.5) (Figures 3A and 3B). Prominent among DEGs showing higher expression were inflammation-, cytochrome-, and lipid metabolism-related genes including *Ifi44* (2.59 logFC), *Irf7* (1.88 logFC), *Cyp17a1* (1.79 logFC), *Cyp3a59* (1.62 logFC), *Elovl5* (1.24 logFC), and *Srebf1* (1.16 logFC) (Figures 3C and S1A). Reduced expression after 12-mon HGI diet was evident for genes encoding several metabolic regulatory proteins, including ornithine aminotransferase (*Oat*, −0.88 logFC), thioredoxin-interacting protein (*Txnip*, −0.74 logFC), and *Pfkfb3* (−1.29 logFC) (Figures 3C and S1). We noted dysregulation of a number of cytochrome P450 family genes, indicating metabolic shifts after 12-mon HGI diet. Consistent with findings after 1-mon HGI diet, GSEA analysis showed upregulation of specific immune- and inflammation-related pathways and of triglyceride metabolism in 12-mon HGI group (Table S2), whereas respiratory electron transport and interleukin-7 signaling pathways were downregulated.

We then focused on the analysis of fatty acid elongation Kyoto Encyclopedia of Genes and Genomes (KEGG) pathway, uncovering several genes showing higher expression with long-term HGI diet including the *Elovl* and *Scd* genes (Figures 3D and S1C). Transcriptomic trends in genes encoding enzymes associated with metabolism of saturated fatty acids (SFAs), monounsaturated fatty acids (MUFAs), and polyunsaturated fatty acids (PUFAs) were consistent in both 1- and 12-mon HGI groups (Figures 3E and S1C). Furthermore, genes for fatty acid binding proteins were also overexpressed in both HGI feeding (Figure S1C and Table S1). However, peroxisome-proliferator activated receptor (PPAR) signaling pathway, upstream of lipid metabolism, did not reveal statistically significant differences.

### Transcriptomic changes in the retina in response to dietary hyperglycemia

**Figure 4:**
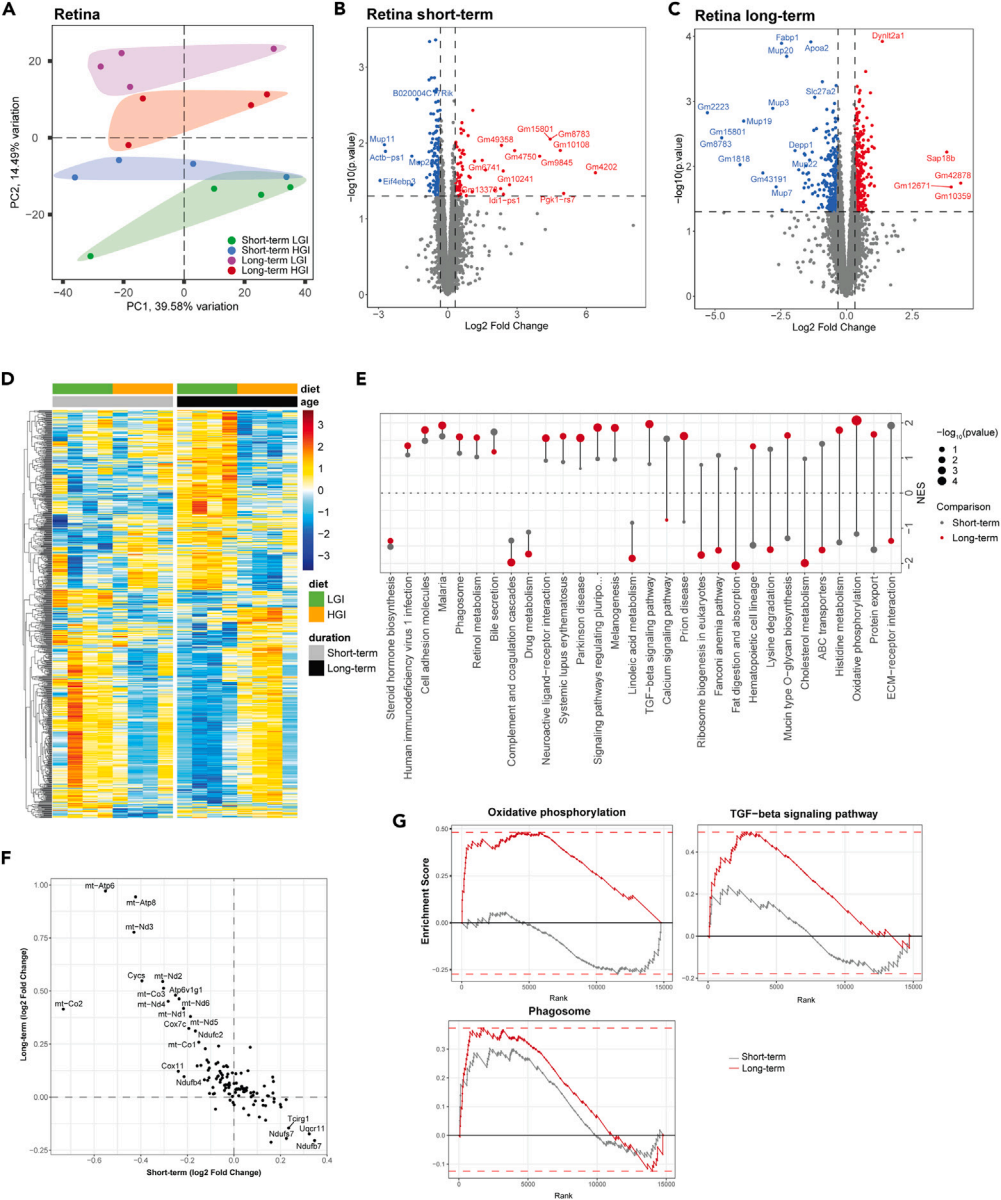
Retinal pathways respond to HGI diet in a duration-dependent manner (A) PCA analysis, colored by retinal age/diet group, shows distinct clustering patterns. (B) Volcano plot showing differentially expressed genes in the retina after short-term HGI and LGI. Horizontal dashed lines represent a p value of 0.05 and vertical dashed lines represent an absolute fold change ≥1.25. (C) Volcano plot for long-term HGI vs. LGI contrast of retinal transcriptomes. (D) Heatmap showing expression trends among retinal all reported DEGs. (E) Pathway enrichment comparison of notable pathways in the retina of short- or long-term HGI-fed animals. Significant KEGG pathways (p < 0.10 in at least one length of exposure matched HGI vs. LGI comparison) are displayed on the y axis and normalized enrichment scores (NESs) are shown on the x axis. Dot size represents -log10(p value), and color represents age-matched diet comparisons. Pathways are sorted top-to-bottom according to age-matched similarity in NES scores. (F) KEGG pathway for oxidative phosphorylation genes shows diet- and length of treatment-related expression trends in the retina. Axes represent the log_2_(FC) for short-term (one-month HGI) and long-term (12-mon-HGI) HGI vs. LGI comparisons. (G) Enrichment curves for oxidative phosphorylation, TGF-β signaling, and phagosome pathways.

Next, we investigated retinal transcriptomes from age-matched mice fed HGI or LGI (Figure 1C). In PCA analysis, retinal transcriptomes clearly clustered according to diet and age (Figure 4A) as in case of liver but had fewer statistically significant DEGs. We therefore modified our significance threshold to p value <0.05 and absolute FC ≥ 1.25 to identify DEGs and predict potential functional implications. In the 1-month diet group, 244 genes were significantly different between retinas of HGI and LGI fed mice (Figures 4B and Table S3). We detected larger differential expression in the 12-mon diet group with a total of 632 genes in HGI- compared to LGI-fed animals (Figures 4C, 4D, and Table S3). Profiles of DEGs showed that only six DEGs had similar patterns of over- or underexpression between 1- and 12-mon diet groups. GSEA indicated an enrichment of several signaling and metabolic pathways (Figure 4E), with oxidative phosphorylation being the most significant KEGG pathway in the retina, but 1- and 12-mon groups showed opposing enrichment scores (Table S3). We noted lower expression of mitochondrially encoded genes in 1-month HGI retinas but higher expression in 12-mon HGI group (Figure 4F). To test whether changes in mitochondrial gene expression were a result of altered mitochondrial population, we investigated transcription of key regulators of mitochondrial biogenesis and mitophagy but detected no significant difference between the diet groups (Table S3).

In both 1- and 12-mon HGI groups, we observed lower expression of the KEGG gene sets for steroid hormone biosynthesis, complement and coagulation cascades, and linoleic acid metabolism. Enrichment curves for oxidative phosphorylation, phagosome, and transforming growth factor β (TGF-β) signaling revealed higher expression in 12-mon HGI mouse retina (Figure 4G). However, we noted cholesterol metabolism and calcium signaling pathways to have distinct trajectories in 1- versus 12-mon diet groups (Figure 4E).

### Fatty acid profiling reveals markers of fatty liver disease

**Figure 5:**
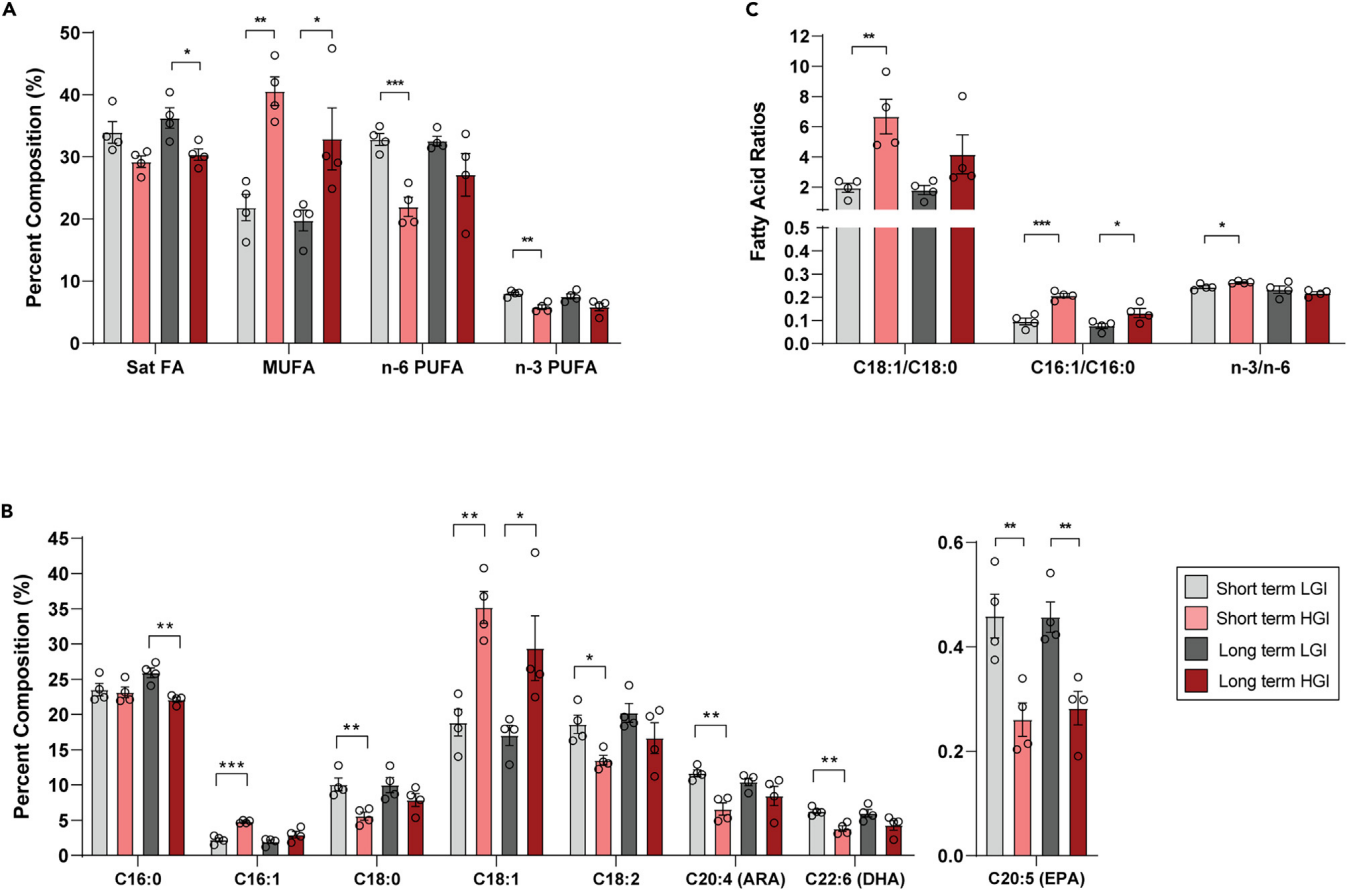
Hepatic fatty acid composition reveals an overabundance of MUFAs and depletion of PUFAs in HGI-fed mice at both ages N = 4 per age/diet group. Individual points represent biological replicates. ∗p < 0.05, ∗∗p < 0.01, ∗∗∗p < 0.001 determined by Student’s *t* test for unpaired samples. Bars represent mean percent composition ±SEM, out of 100%, for (A) and (B). Ratios are presented for (C). (A) Summary statistics for the sums of SFAs, MUFAs, n-6 PUFAs, and n-3 PUFAs. (B) Major fatty acid compositions determined by gas chromatography, colored by age/diet group. (C) Fatty acid ratios for C18:1/C18:0, C:16:1/C:16:0 and n-3/n-6.

To seek biochemical validation of the transcriptomic differences, we profiled fatty acid composition in liver samples. HGI feeding significantly affected key species of SFAs, MUFAs, and PUFAs in the liver (Figure 5). HGI-fed animals had 16.3% lower SFAs compared with LGI-fed animals in the 12-mon group (p < 0.05) (Figure 5A). Specifically, we noted reduced levels of two major SFAs—C16:0 (palmitic acid, p < 0.01 in 12-mon group) and C18:0 (stearic acid, p < 0.01 in 1-month feeding group) (Figure 5B). HGI feeding resulted in 85.8% and 66.6% increase in levels of total MUFAs (p < 0.05) in 1- and 12-mon fed mice, respectively (Figure 5A). C16:1 n-7 (palmitoleic acid) and C18:1 n-9 (oleic acid) were more abundant in HGI livers compared with the LGI diet group (p < 0.05) (Figure 5B). Decreased SFAs combined with increased MUFAs in response to HGI feeding resulted in elevated C18:1/C18:0 and C16:1/C16:0 ratios (Figure 5C).

In addition, overall PUFA content for HGI livers was lower than the LGI group. 1-month HGI diet reduced total n-6 PUFA, C18:2 (linoleic acid), and C20:4 (arachidonic acid) levels in the liver by 33.1%, 27.4%, and 43.7%, respectively, compared with LGI diet (p < 0.05) (Figures 5A and 5B). Total hepatic n-3 PUFA, C22:6 (docosahexaenoic acid - DHA), and C20:5 (eicosapentaenoic acid, EPA) levels were significantly decreased by 27.4%, 39.1%, and 43.2%, respectively, after 1-month HGI feeding compared with LGI (Figures 5A and 5B). Feeding HGI diet for 12-mon resulted in decreased levels of total hepatic n-3 PUFA by 23.4%. The n-3 alterations were composed of a 28.5% and 38.1% lower abundance of DHA and EPA, respectively. PUFA ratio of n-3/n-6 in one-month HGI-fed group was 8.3% higher than that of the LGI-fed group (p < 0.05) (Figure 5C).

### Prolonged HGI feeding results in systemic hypercholesterolemia

**Figure 6:**
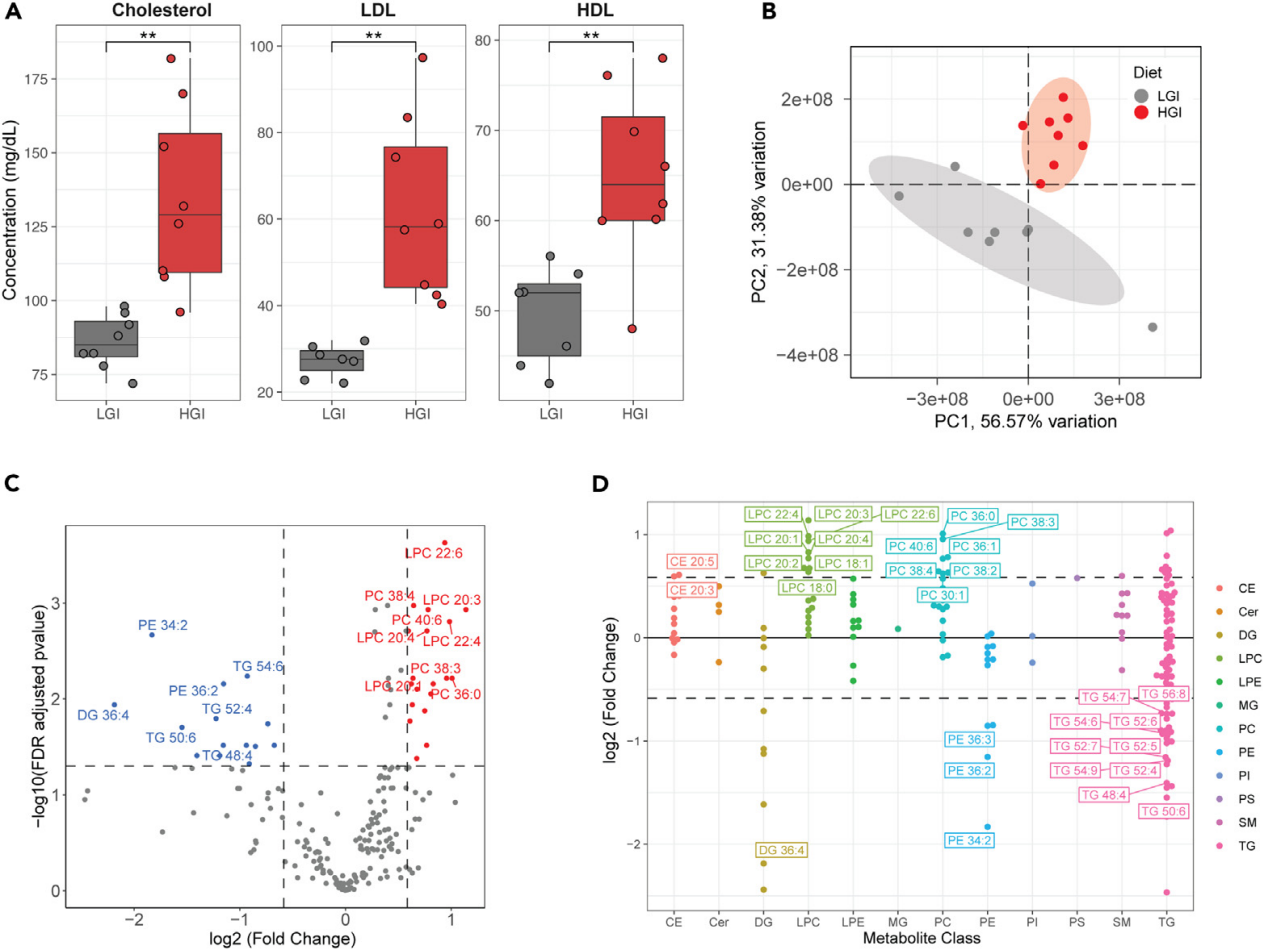
Altered blood plasma metabolites in response to prolonged HGI feeding (A) Blood plasma total cholesterol, LDL, and HDL in twelve-month-diet mice, colored by diet. Significantly elevated total cholesterol, LDL, and HDL indicate hypercholesterolemia in the mice fed the HGI diet. ∗∗p < 0.01 using Student’s *t* test with N = 8 mice per diet group. (B) Blood plasma PCA partial least-squares regression plot using metabolomics data generated from a C8 lipidomics column. (C) Differentially abundant blood plasma metabolites. Horizontal dashed lines represent an FDR-adjusted p value of 0.05, and vertical dashed lines represent an absolute log2(FC) ≥ 1.5. (D) Differentially abundant metabolites grouped by metabolite class. Horizontal dashed lines represent an absolute log2(FC) ≥1.5 in HGI vs. LGI. Text labels represent individual metabolites. From left to right: CE, Cholesterol Ester; Cer, Ceramide; DG, Diglyceride; LPC, Lysophosphatidylcholine; LPE, Lysophosphatidylethanoamine; MG, Monoglyceride; PC, Phosphatidylcholine; PE, Phosphatidylethanolamine; PI, Phosphatidylinositol; PS, Phosphatidylserine; SM, Sphingomyelin; TG = Triglyceride.

We checked plasma lipids from HGI- and LGI-fed mice to measure the systemic impact of diet-related adaptations. Due to sample limitations, this analysis was conducted only on the 12-mon groups (Figure 1B). Relative to LGI, HGI feeding resulted in a 56.4% increase in total plasma cholesterol, a 129% increase in LDL, and a 31.5% increase in HDL (p < 0.01) (Figure 6A and Table S4). Total plasma triglycerides did not show significant abundance change in this experiment (39.3 ± 2.1 mg/dL in LGI vs. 35.5 ± 2.6 mg/dL in HGI; p = 0.28). Further, we performed metabolomic analysis on blood plasma using a C8 lipidomics liquid chromatography-tandem mass spectrometry (LC/MS-MS) column. Using partial least-square regression, the metabolomes of HGI and LGI fed mice displayed distinct clustering (Figure 6B). Compared to the LGI-fed group, the HGI-fed animals had 16 metabolites at significantly lower concentrations, and 19 metabolites at higher concentrations (Figures 6C and Table S5). By separating plasma metabolites by class, we identified a trend of over-abundant phosphatidyl choline (PC) and lysophosphatidyl choline (LPC) with a marked decrease in specific triglyceride species in HGI-fed mice compared to LGI (Figure 6D).

### Diet-dependent and aging-associated alterations in liver and retina transcriptomes

**Figure 7:**
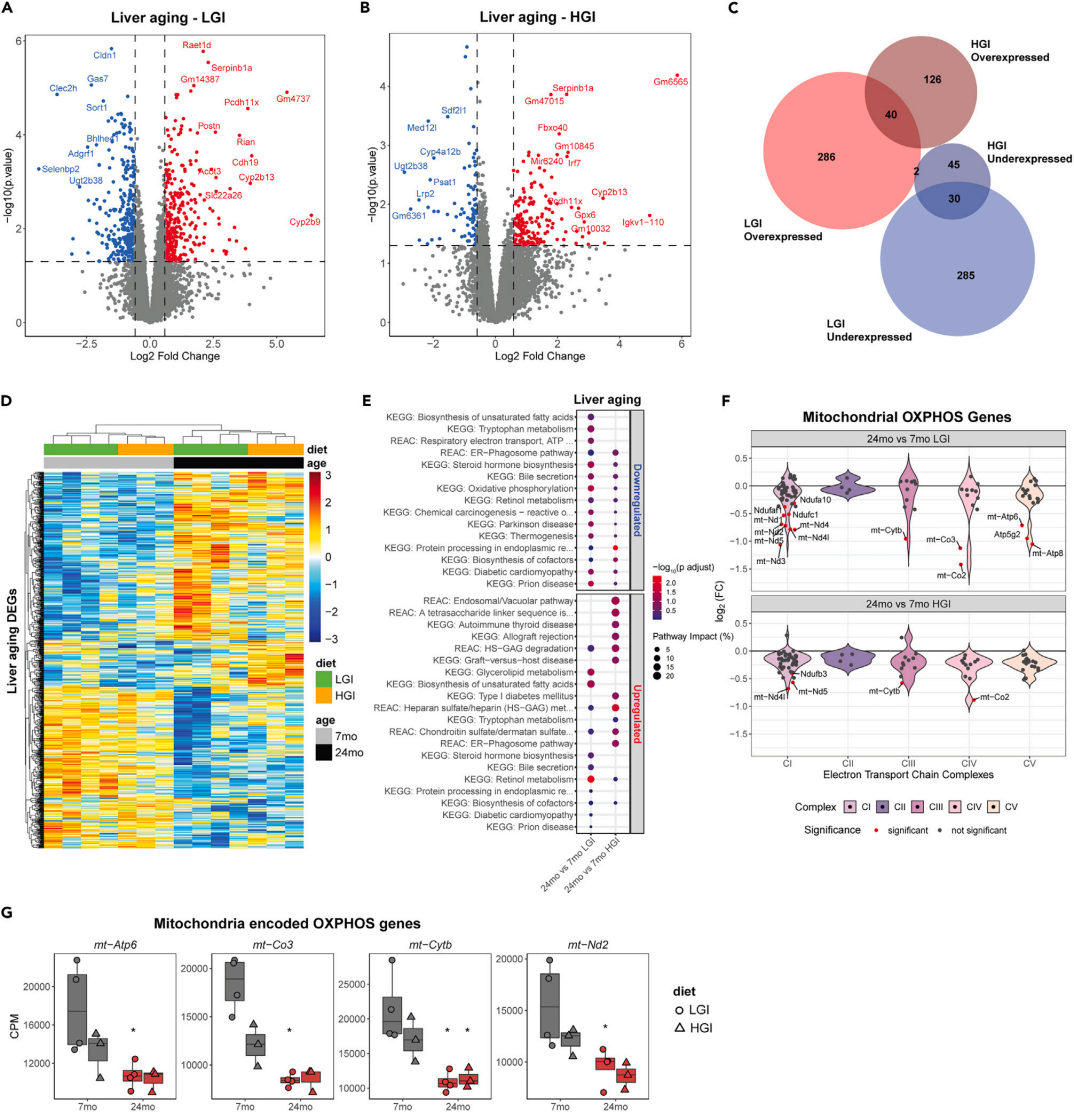
Diet-aging interaction influences hepatic expression of oxidative phosphorylation genes (A) Volcano plot showing aging-related DEGs in livers of LGI fed animals. (B) Volcano plot of DEGs in livers of HGI-fed animals. (C) Overlap of hepatic aging-related DEGs from LGI and HGI groups. (D) Heatmap of all liver DEGs from aging comparisons of HGI and LGI groups. (E) Pathway mapping of hepatic aging-related DEGs from the two diet groups. (F) Mitochondrial OXPHOS genes change expression in liver with age and diet. Significant DEGs are highlighted in red. (G) Mitochondria-encoded oxidative phosphorylation genes are uniquely affected in the liver by aging and GI exposure. ∗ denotes whether a gene is statistically significant (p < 0.05) within a diet group aging comparison.

To examine the impact of dietary GI on aging, we performed differential expression analysis between young and old animals within each diet group (Figure 1C). Liver comparisons identified 644 aging-related DEGs in the LGI-fed animals, whereas the HGI group had 244 DEGs differential with aging (Figures 7A and 7B). LGI aging was associated with higher expression of *Cyp2b9* (6.37 logFC) and dysregulation of several other cytochrome P450 genes. Interestingly, *Ppara* (0.72 logFC) and its downstream genes for acyl-CoA thioesterases (*Acot3* 2.6 logFC, *Acot1* 2.18 logFC, *Acot2* 2.15 logFC, *Acot4* 1.35 logFC) were expressed at higher levels in livers of older LGI mice (Figures 7B and Table S2). *Ppara* and *Acot* genes suggest activity in lipid metabolism and intracellular levels of fatty acids in livers of LGI-fed animals. In addition, key glucose homeostasis and metabolic regulator *Foxo1* was expressed at higher levels (0.59 logFC) in LGI aging of the liver. Among genes showing lower expression in aging comparisons, *Selenbp2* (−4.38 logFC), a target of peroxisome proliferators and linked with *Ppara* transcription, had the highest change between old and young LGI livers. In addition, multiple *Elovl* family genes (*Elovl3* -2.43 logFC, *Elovl5* -1.38 logFC, *Elovl2* -0.62 logFC) were expressed at lower levels in LGI aging (Table S2).

Strikingly, less than 10% of the aging-related DEGs were common between LGI and HGI groups (Figure 7C), corroborating major shifts due to dietary GI.^31^ Distinct expression trends of aging-related DEGs were also evident in the two groups (Figure 7D). Pathway analysis of DEGs in aging comparisons revealed significant disruption in key fatty acid oxidation and metabolic pathways and suggested reduced expression of the respiratory electron transport in both LGI and HGI groups (Figures 7E and Table S2). Aging livers in the HGI group exhibited higher expression of inflammation-related genes, *Igkc* (3 logFC) and *Irf7* (2.3 logFC). Older HGI animals showed higher expression of *Scd2* (1.45 logFC), a fatty acid desaturase, a trend reported in liver diseases.^33^ The genes showing lesser expression included insulin receptor substrate 1 (*Irs1*, -0.89 logFC) and several oxidative phosphorylation (OXPHOS) genes (Table S2). GSEA revealed positive enrichment of several inflammation pathways, whereas core metabolic processes like the citric acid cycle (TCA) and OXPHOS were negatively enriched (Table S2). Closer evaluation of the mitochondrial OXPHOS pathway revealed lower expression of leading-edge genes that are part of the electron transport chain complexes (Figure 7F). Additionally, several mitochondria-encoded OXPHOS genes were expressed at lower levels in HGI aging (Figure 7G).

Aging-related transcriptomic trends in the retina were distinct for LGI and HGI diet groups in PCA analysis (Figure S2A). Comparison of retinal transcriptomes from old and young animals group (Figure 1C) revealed substantially higher expression of the retinol dehydrogenase gene Rdh1 (1.6 logFC) and several complement factors in the LGI group (Figures S2B and S2C). In LGI aging, OXPHOS genes were underexpressed (Table S3) along with TCA enzymes and associated factors such as *Dlat* (−0.33 logFC). GSEA analysis of LGI aging comparisons revealed downregulation of calcium signaling pathways with calmodulin genes featured in the leading edge (Table S3). Retinal aging in the HGI group was notable because of dysregulated expression of 303 genes including differential expression of *Rdh1* (1.66 logFC), complement genes, and *Rgr* (1.5 logFC) (Figures S2C, S2D, and Table S3). As many as 111 DEGs had analogous expression trends in HGI and LGI aging comparisons, suggesting their diet-independent role in retinal aging (Figure S2E). Similar to LGI, several complement factor genes show higher expression with age in the retina of HGI-fed animals (Figure S2C). GSEA analysis using KEGG and Reactome databases revealed overlapping pathways mapping for overexpressed genes from LGI and HGI aging comparisons. Aging-related underexpressed genes in retina of LGI- and HGI-fed animals mapped to distinct pathways (Figure S2F). The respiratory electron transport pathway (in Reactome pathway database; https://reactome.org/userguide/pathway-browser) was only impacted in LGI aging, whereas extracellular matrix reorganization was affected by HGI aging. Given the link of advanced age with retinal diseases, we checked expression levels of retina disease genes (RetNet, https://sph.uth.edu/RetNet/) and detected 10 DEGs in 24-mon-old retinas, mostly within the HGI group (Figure S2G).

## Discussion

Preventative strategies based on lifestyle modifications are essential tools for managing and alleviating non-communicable diseases (NCDs), such as AMD. Healthy diets appear to offer opportunities in global efforts to limit health burdens imposed by obesity and diabetes, among other NCDs, that have reached epidemic proportions.^1^ Decades of research characterizing health impacts of typical dietary fats and high-fat diets have led to guidelines and policy changes to avert NCDs.^34^ Dietary glycemic exposure directly influences postprandial blood glucose and is key for controlling risks for metabolic diseases related to hyperglycemia.^35^^,^^36^ Data on GI of food items can help in controlling dietary glycemic exposure. Here, we report the responses of two metabolically and developmentally different tissues—the liver and the retina—to high and low GI diets for short or prolonged periods, and at different ages. Our transcriptome-wide analysis revealed dietary GI-associated shifts in metabolic pathways in both the liver and the retina, and inflammation processes upon short- and long-term HGI feeding in the liver. Hepatic fatty acid profiles matched transcriptomic findings and revealed correspondent changes in saturated and unsaturated fatty acid abundances linked to glycemic exposure. Moreover, we noted diet-aging interaction influencing tissue-specific aging in both tissues. Our data detail tissue-specific and length of exposure-dependent adaptations to dietary glycemic exposure that may be helpful toward mitigation strategies for better health.

The liver functions at the crossroads of the endocrine and gastrointestinal systems. It performs vital metabolic regulation including glycemic control by modulating glycolysis/gluconeogenesis and cholesterol metabolism.^11^^,^^21^ In comparison with prior experiments, using plasma and urine, demonstrating transitions from sugar to lipid metabolism in older mice on prolonged HGI diets, here we observe a rapid shift in fatty acid and carbohydrate metabolism processes as early as 1-mon of HGI feeding. Overexpressing genes *Rela* (component of the NF-κB machinery) and *Gpi1* indicate hepatic inflammation and altered metabolic state.^37^^,^^38^^,^^39^ Pathway changes in short-term HGI-fed diet suggest hepatic distress that potentially triggers *ApoA4* expression for its hepatoprotective effects.^40^ ApoA4 is reported to counter diet-induced hepatic steatosis,^41^ and its immediate activity in livers of HGI fed-animals indicates detrimental effects even in short-term exposures. Lower expression of cholesterol 7α-hydrolase (*Cyp7a1*), which constitutes a rate-limiting step of bile acid synthesis from cholesterol, is consistent with cholesterol accumulation under short-term HGI feeding.^42^

Long-term HGI feeding enhanced transcriptomic dysregulation in fatty acid metabolism and inflammation pathways. Elevated expression of fatty acid elongase-5 (*Elovl5*) and its transcriptional regulator sterol regulatory element binding transcription factor 1 (*Srebf1*) was pronounced in livers of long-term HGI-treated animals. *Srebf1* is induced by insulin and in turn activates *Elovl5* to increase biosynthesis of unsaturated fatty acids or to redirect lipid abundance.^43^^,^^44^^,^^45^ Long-term HGI-induced activation of lipid biosynthesis genes may represent the liver’s adaptive mechanism for countering excess dietary glucose by increasing conversion of glucose to fatty acids.^21^^,^^46^ Shifts in liver fatty acid composition substantiate transcriptomic findings and show significantly increased MUFAs and lower levels of PUFAs with HGI diet. These changes in hepatic fatty acid content largely resemble fatty acid changes seen in non-alcoholic fatty liver disease^47^ and agree with previous reports of hepatic steatosis in animals fed with HGI diets.^48^ Interestingly, many significant genes for fatty acid metabolism and inflammation pathways were also observed to be significant in mice fed a high-fat diet.^49^ Unlike the high-fat diet, which has significantly increased calories per gram, our HGI diet was isocaloric to the LGI diet, implying that rapid release from dietary sources of sugar into the bloodstream and tissues alone is enough to alter hepatic transcriptome, lipid composition, and systemic cholesterol levels. The diet regimens employed in this study captures the importance of carbohydrate quality in maintenance of health; however, it does not recapitulate the full extent and heterogeneity of the Western diet, which also contains excessive saturated fats and other food additives, a subject for future studies.

Phototransduction is an energetically demanding process making the retina a highly metabolic organ.^50^ Glucose metabolism is essential for retinal homeostasis, and there are distinct metabolic requirements making it vulnerable to improper metabolite supply.^23^^,^^51^ Links between dietary glycemic exposure and diseases including AMD and diabetic retinopathy emphasize the metabolic vulnerability of the retina.^6^^,^^31^ Our diet experiments showed retinal response to dietary GI depended on duration of feeding. As in case of the liver, HGI diet was linked with metabolic changes in the retina. An interesting HGI-linked trend was noted in gene expression of retinal mitochondrial genes. Genes coding for subunits of the electron transport chain were distinctly overexpressed in retinas of long-term HGI animals and showed reduced expression in short-term diet treatment. Mitochondria have significant influence on retinal health, disease, and aging,^52^^,^^53^ and its response to dietary glycemic exposure warrants further investigation. Retinal adjustment to diet solicits lifestyle mitigation strategies for vision disorders.

Our study design has strengths and weaknesses. The key strengths are the use of individually housed animals and well-defined macronutrient-matched diets that lead to well characterized phenotypic outcomes and clear contrasts between diet, tissue, age, and exposure time. The limitations include the use of male mice only, two different cohorts of mice, and use of a diet based on the AIN-93G semipurified diet and not chow-based diets. Future investigations will evaluate the impact of diet and aging in genetic disease models and in multiple tissues with both males and females by adding high-throughput proteome and metabolome assays to complement the initial transcriptome focus. In summary, this study elucidates molecular mechanisms underlying tissue-specific adaptation to dietary GI and helps identify pathogenic links of HGI diets in the liver and retina. It will be of interest to extend this study design to other tissue systems and diets to uncover the network of diet- and nutrition-dependent adaptations, especially with aging in combination with additional “omics” assays.

### Limitations of the study

Despite the rigor employed, this study has a few limitations. By focusing on male animals only, we miss on whether and how sex may influence response to diet. A diet study such as this should ideally include mice derived from a single cohort with treatments starting at the same time. Finally, in addition to transcriptome and lipid landscapes, characterizing the epigenomic and protein as well as metabolite profiles will provide comprehensive insights into time-dependent and tissue-specific adaptations to diet.

## STAR★Methods

### Key resources table

**Table:** 

REAGENT or RESOURCE	SOURCE	IDENTIFIER
**Critical commercial assays**		

AllPrep DNA/RNA Micro Kit	Qiagen	Cat# 80284
TruSeq Stranded mRNA Sample Prep Kit	Illumina	Cat# 20020595
RNA Screentape	Agilent Technologies	Part# 5067-5576
Methanol	MilliporeSigma	Cat# 179337
Hexane	MilliporeSigma	Cat# 32293
acetyl chloride	MilliporeSigma	Cat# 00990
FAME reference standard	Nu-Chek Prep	Cat# GLC462
Gas Chromatograph	Shimadzu Scientific Instruments	Cat# Nexis GC-2030
SLB®-IL111 Capillary GC Column	MilliporeSigma	Cat# 28927-U
Cholesterol assay	Olympus	AU480 Procedural Insert (OSR6116, OSR6216)
Triglycerides assay	Olympus	AU400 Procedural Insert (OSR6033, OSR6133)
HDL-cholesterol assay	Olympus	AU480 Procedural Insert (OSR6195, OSR6295)

**Deposited data**		

Raw and analyzed data	This paper	GEO: GSE243843

**Experimental models: Organisms/strains**		

Mouse: C57BL/6J, Male	The Jackson Laboratory	N/A

**Software and algorithms**		

kallisto	Ref.^54^	https://github.com/pachterlab/kallisto
tximport	Ref.^55^	https://bioconductor.org/packages/release/bioc/html/tximport.html
R version 4.2.0	The R project	https://www.r-project.org/
edgeR	Ref.^56^	https://bioconductor.org/packages/release/bioc/html/edgeR.html
limma	Ref.^57^	https://bioconductor.org/packages/release/bioc/html/limma.html
gProfiler	Ref.^58^	https://biit.cs.ut.ee/gprofiler/gost
tidyverse	N/A	https://www.tidyverse.org/
pheatmap	Raivo Kolde	https://cran.r-project.org/web/packages/pheatmap/index.html

### Resource availability

#### Lead contact

Further information and requests for resources and reagents should be directed to the lead contact, Anand Swaroop (swaroopa@nei.nih.gov). This study did not generate new unique reagents or code.

#### Materials availability

This study did not generate new unique reagents.

#### Data and code availability

• Data: Sequencing data have been deposited at NCBI GEO (GSE243843) and are publicly available as of the date of publication. Accession numbers are also listed in the key resources table.
• Code: This paper does not report any original code.
• Any additional information necessary to reproduce results or reanalyze the data presented in this study will be made available upon request to the lead contact.

### Experimental model and study participant details

All animal work was performed at the Tufts University Human Nutrition Research Center on Aging (HNRCA) and approved by the Tufts University Institutional Animal Care and Use Committee (IACUC) in accordance with the Statement for the Use of Animals in Ophthalmic and Vision Research of the Association for Research in Vision and Ophthalmology. Male C57BL/6J mice at either 6- or 10- months of age were obtained from the aging colony at the Jackson Laboratory at (Bar Harbor, ME). Mice were individually housed and maintained on a 12 h/12 h light/dark cycle (7:7) at 24°C and 40% humidity.

### Method details

#### Experiment design

Animals were split into a total of four treatment groups (n = 4), per age and per diet. Low (LGI) and high glycemic index (HGI) diets were isocaloric with identical macronutrient compositions, while only varying dietary starch composition (Figure 1 and Table S1). The macronutrient composition of our diets is based on the AIN-93G semipurified diet. The LGI diet consisted of 70% amylose and 30% amylopectin (Hylon VII starch, Ingredion Inc., Bridgewater, NJ), and the HGI diet consisted of 100% amylopectin (Amioca starch, Ingredion Inc., Bridgewater, NJ). Diets were formulated by Bio-Serv (Frenchtown, NJ). Mice were pair-fed to ensure equal consumption in both HGI and LGI diet groups. 6-month-old mice were aged on experimental diets for a duration of one month until reaching 7-month-old of age. 10-month-old mice were fed a standard chow diet (Teklad 7012, Harlan Laboratories, Madison, WI) *ad libitum* for a duration of 2 months until 12-month-old of age. 12-month-old mice were then aged for twelve months on experimental diets, reaching 24-month-old. Mice were weighed on a weekly basis throughout the course of the study. At the end of each treatment, mice were fasted at 8 a.m. for 6 h and euthanized at approximately the same time of day, 2 p.m.–4 p.m. Whole blood was collected via cardiac puncture into tubes containing EDTA (ethylenediaminetetraacetic acid) at the time of euthanasia and all tissues were collected. Blood was centrifuged at 2000 x g for 10 min. Supernatant was collected, aliquoted, and stored at −80°C. Plasma lipids were evaluated via clinical chemistry analyzer (BeckmanCoulter, Brea, CA) by the Nutrition Evaluation Laboratory at the HNRCA.

#### RNA isolation and sequencing

Total RNA was isolated from samples using a RNeasy Mini Kit according to the manufacturer’s instructions (Qiagen, Hilden, Germany). Briefly, 100mg of liver tissue or whole retinas were homogenized and RNA-seq libraries prepared using a TruSeq Stranded mRNA Sample Prep Kit (Illumina, San Diego, CA, USA) as previously described.^59^^,^^60^ Libraries were pair-end sequenced to 101 bases on an Illumina HiSeq 2500 system. All RNA-seq samples selected for downstream analysis had a sequencing depth greater than 10 million reads.

#### Fatty acid composition analysis

Hepatic lipids were extracted by homogenizing tissues samples in a methanol/hexane solution (4:1 v/v) with butylated hydroxytoluene as an antioxidant as described previously.^61^ Fatty acids were converted to fatty acid methyl esters (FAME) by reaction with acetyl chloride at 100°C for 1 h and the resulting FAME separation was performed by gas chromatography on a Shimadzu GC2030 (Shimadzu Scientific Instruments, Columbia, MD) equipped with a flame ionization detector (FID) and a fused-silica capillary SLB-IL111 column (30 m × 0.25 mm, φ0.2 μm; Sigma Aldrich). Helium was used as carrier gas. Peaks were identified by comparison to retention times of a reference mixture (Nu-Chek Prep. Inc., Elysian MN). Data were presented as relative percentages of total fatty acids (% wt). Graphing and statistical testing using multiple two-sided t tests were performed using Graphpad Prism 9 for Windows (Graphpad Software, La Jolla, California USA).

#### Blood metabolomics

Plasma lipids were profiled using a Shimadzu Nexera X2 U-HPLC (Shimadzu Corp.; Marlborough, MA). Lipids were extracted from plasma (10 μL) using 190 μL of isopropanol containing 1,2-didodecanoyl-*sn*-glycero-3-phosphocholine (Avanti Polar Lipids; Alabaster, AL). After centrifugation, supernatants were injected directly onto a 100 × 2.1 mm, 1.7 μm ACQUITY BEH C8 column (Waters; Milford, MA). The column was eluted isocratically with 80% mobile phase A (95:5:0.1 v/v/vol 10mM ammonium acetate/methanol/formic acid) for 1 min followed by a linear gradient to 80% mobile-phase B (99.9:0.1 v/v methanol/formic acid) over 2 min, a linear gradient to 100% mobile phase B over 7 min, then 3 min at 100% mobile-phase B. MS analyses were carried out using electrospray ionization in the positive ion mode using full scan analysis over 200–1100 m/z at 70,000 resolution and 3 Hz data acquisition rate. Other MS settings were: sheath gas 50, in source CID 5 eV, sweep gas 5, spray voltage 3 kV, capillary temperature 300°C, S-lens RF 60, heater temperature 300°C, microscans 1, automatic gain control target 1e6, and maximum ion time 100 ms. Lipid identities were denoted by total acyl carbon number and total number of double bond number.

### Quantification and statistical analysis

#### Sequencing data analyses

Raw fastq files from the Illumina HiSeq 2500 System were quality checked and processed following standard parameters.^59^ Briefly, reads were aligned to the v104 *Mus musculus* Ensembl gene annotation. Gene counts matrices were normalized and expressed in counts per million (CPM) using the edgeR package from Bioconductor.^62^ To remove lowly expressed genes, a filter was used to select for genes with an average expression of greater than 1 CPM in any experimental group. To account for index hopping arising from sequencing liver and retina samples on the same lane, 29 highly expressed retinal genes, which comprised the highest loading score values from principal component analysis, were removed from the liver samples.

Differential expression analysis comparing HGI relative to LGI groups was conducted using the voom function on the normalized, filtered counts and fitted to a linear model with the limma Bioconductor package.^57^ A threshold criteria of p value of <0.05 with an absolute fold change of ≥1.5 for liver comparisons and ≥1.25 for retina comparisons were used to define genes as differentially expressed. Pathway enrichment of differentially expressed genes was conducted using the gprofiler2 (v0.2.0) package.^63^^,^^58^ KEGG, Reactome, and GO:BP gene sets were used for pathway enrichment of differentially regulated genes in the liver. Gene set enrichment analysis (GSEA) was performed using the fgsea (v1.18.0) package, with genes ranked by log_2_ fold change.^64^ KEGG gene sets from gprofiler2 were used for visualizing enrichment curves using custom scripts for retina comparisons. Additional R packages included ggplot2, PCAtools, ggpubr, and pheatmap.

#### Miscellaneous statistical analysis and data visualization

Unless otherwise specified, the R programming environment and tidyverse packages were used for analysis and visualization.

## Supplemental information

Document S1. Figures S1 and S2 and Tables S1, S4, and S5

Table S2. Differential gene expression and pathway analysis in liver samples from HGI- and LGI-fed animals, related to Figures 2, 3, and 7 Submitted as Excel Data File.

Table S3. Differential gene expression and pathway analysis in retina samples from HGI and LGI fed animals, related to Figures 4 and 7 Submitted as Excel Data File.
