# Supplementary material for: Selective transcriptomic dysregulation of metabolic pathways in liver and retina by short- and long-term dietary hyperglycemia
Source: iScience. 2024 Jan 19;27(2):108979. doi: 10.1016/j.isci.2024.108979 (PMC10850775; doi:10.1016/j.isci.2024.108979)
Supplement: Document S1. Figures S1 and S2 and Tables S1, S4, and S5 [file mmc1.pdf]

iScience, Volume *27*

Supplemental information

Selective transcriptomic dysregulation of metabolic pathways in liver and retina by short- and long-term dietary hyperglycemia

Anupam K. Mondal, Daniel C. Brock, Sheldon Rowan, Zhi-Hong Yang, Krishna Vamsi Rojulpote, Kelsey M. Smith, Sarah G. Francisco, Eloy Bejarano, Milton A. English, Amy Deik, Sarah Jeanfavre, Clary B. Clish, Alan T. Remaley, Allen Taylor, and Anand Swaroop

Figure S1

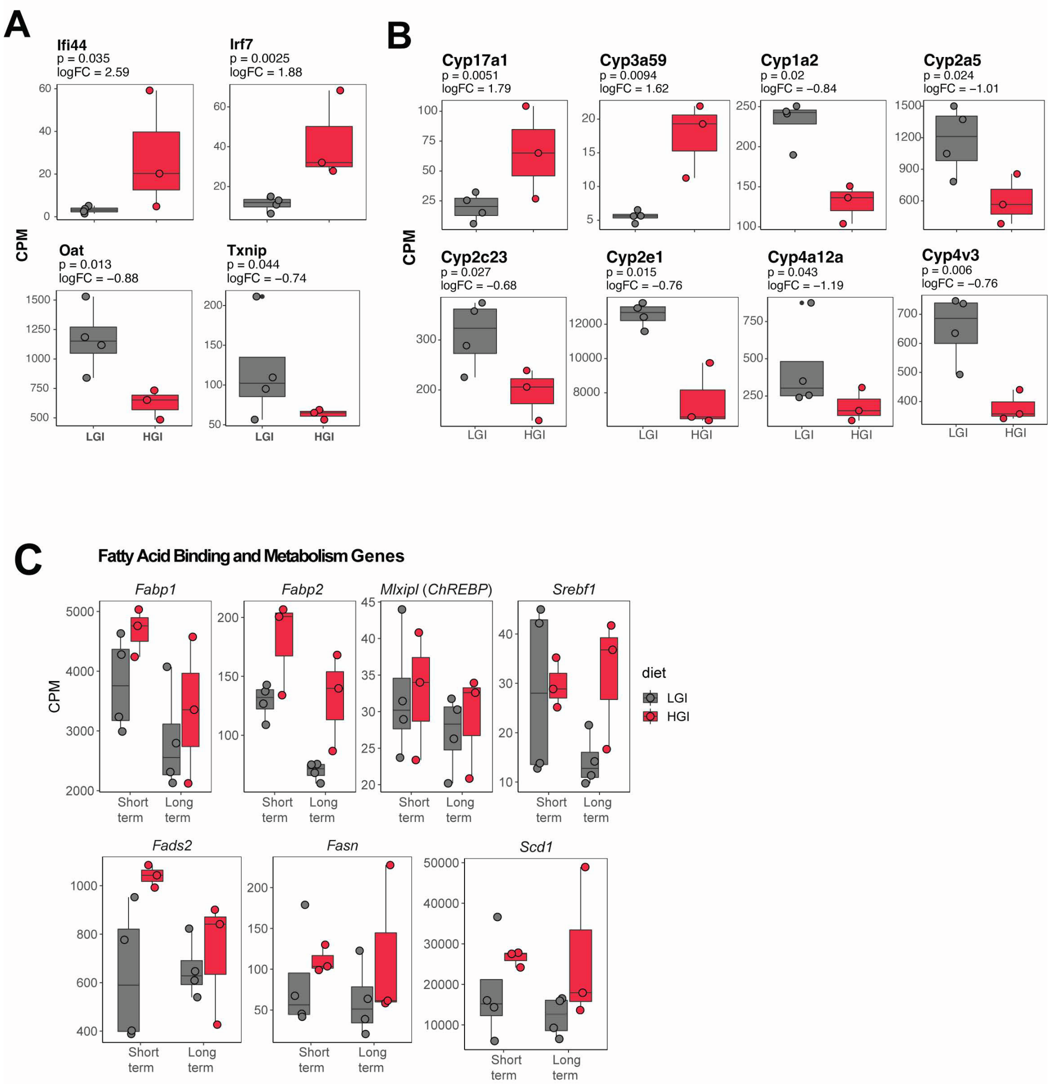

Figure S1. Hepatic adaptation to long-term HGI feeding involves realignment of inflammation and metabolic regulators (Related to Figure 2 and Figure 3).

(A) Inflammation and metabolic associated DEGs affected by long-term HGI diet.

(B) Several cytochrome P450 enzymes that play essential roles in liver metabolism are sign ificantly dysregulated upon chronic HGI treatment.

(C) Expression of genes encoding regulators of fatty acid binding and metabolism.

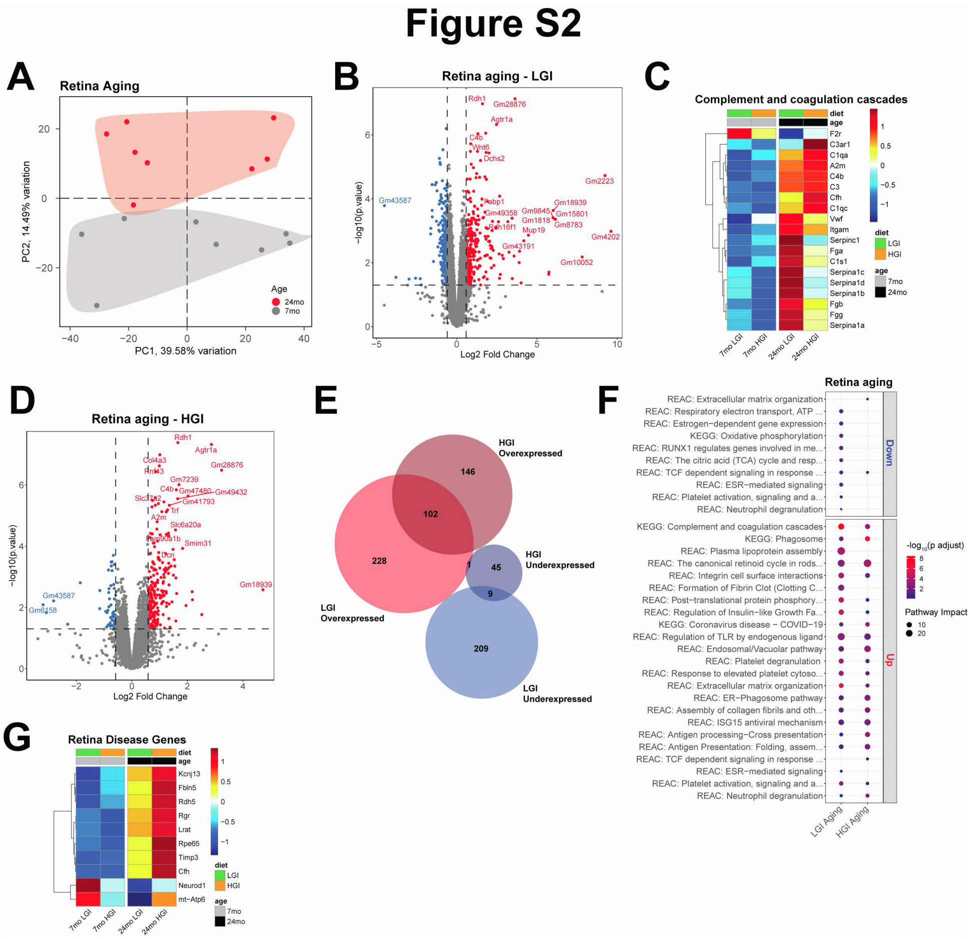

Figure S2. Retinal diet-aging interaction impacts oxidative phosphorylation and complement pathways (Related to Figure 7).

(A) Principial component analysis displays clustering of aging retina transcriptomes in both LGI and HGI groups.

(B) Volcano plot of aging linked retinal DEGs in the LGI group.

(C) Heatmap of complement and coagulation cascade related genes in retinas of LGI and HGI animals.

(D) Volcano plot of aging linked retinal DEGs in the HGI group.

(E) Venn diagram showing overlap among retinal aging genes in the two GI treatments.

(F) Pathway analysis of retinal DEGs in aging comparisons of LGI and HGI groups.

(G) Transcriptomic trends of retinal disease genes that are significantly differentially expressed with respect to aging and GI treatments.

Table S1. Composition of high glycemic (HGI) and low glycemic (LGI) index diets (Related to Figure 1).

	HGI diet	LGI diet
Composition (% kcal)		
Carbohydrate	65	65
Protein	21	21
Fat	14	14
Ingredients (g/kg)		
Amioca starch	542	0
Hylon VII starch	0	542
Casein	200	200
Sucrose	85	85
Soybean oil	56	56
Wheat bran	50	50
Dl-Methionine	2	2
Vitamin mix	10	10
Mineral mix	35	35

Table S4. Abundance of total cholesterol, HDL and triglycerides in blood samples of long-term HGI and LGI fed animals (Related to Figure 6).

Mouse ID	Treatment	Cholesterol	HDL	Triglycerides	LDL
**1**	HGI	132	66	42	57.6
**2**	HGI	170	78	42	83.6
**3**	HGI	182	76	44	97.2
**4**	HGI	126	60	36	58.8
**7**	HGI	108	62	28	40.4
**9**	HGI	110	60	26	44.8
**12**	HGI	152	70	38	74.4
**13**	HGI	96	48	28	42.4
**17**	LGI	88	52	36	28.8
**19**	LGI	98	56	50	32
**25**	LGI	96		38	
**26**	LGI	78	42	42	27.6
**29**	LGI	72	44	30	22
**30**	LGI	82	52	36	22.8
**31**	LGI	92	54	38	30.4
**32**	LGI	82	46	44	27.2
	T-test	0.000794	0.002437	0.27924	0.000737

Table S5. Differentially abundant plasma metabolites of long-term HGI vs LGI fed mice (Related to Figure 6).

HMDB_ID	HMDB_ID_ specificty (1=match; 2=representative)	Metabolite	FC	log2 FC	p.adjusted	Column
HMDB0010404	1	LPC 22:6	1.9173	0.93911	0.00012058	C8
HMDB0008048	2	PC 38:4	1.5627	0.64408	0.0007212	C8
HMDB0010393	2	LPC 20:3	2.2023	1.139	0.00077091	C8
HMDB0010401	1	LPC 22:4	1.978	0.98405	0.00077091	C8
HMDB0008057	2	PC 40:6	1.7186	0.7812	0.00077091	C8
HMDB0006736	2	CE 20:3	1.5102	0.59471	0.00089723	C8
HMDB0010395	1	LPC 20:4	1.7061	0.7707	0.0017019	C8
HMDB0008928	2	PE 34:2	0.28199	-1.8263	0.0019008	C8
HMDB0008047	2	PC 38:3	1.94	0.95609	0.0028241	C8
HMDB0005391	2	TG 54:6	0.52497	-0.92969	0.005014	C8
HMDB0007248	2	DG 36:4	0.21964	-2.1868	0.005217	C8
HMDB0008036	2	PC 36:0	2.0115	1.0083	0.005217	C8
HMDB0010391	1	LPC 20:1	1.777	0.82943	0.005217	C8
HMDB0010384	1	LPC 18:0	1.5563	0.63809	0.005217	C8
HMDB0007870	2	PC 30:1	1.5403	0.62325	0.005217	C8
HMDB0042789	2	TG 48:5	0.20436	-2.2908	0.0060143	C8
HMDB0008994	2	PE 36:2	0.44944	-1.1538	0.0060143	C8
HMDB0010392	1	LPC 20:2	1.5977	0.676	0.0066356	C8
HMDB0008270	2	PC 38:2	1.5495	0.63184	0.0066356	C8
HMDB0001893	1	alpha-Tocopherol	1.7498	0.80715	0.0067063	C8
HMDB0011241	2	PC P-36:0 or PC O-36:1	1.6811	0.7494	0.0070942	C8
HMDB0005363	2	TG 52:4	0.42778	-1.2251	0.0090591	C8
HMDB0010497	2	TG 50:6	0.34206	-1.5477	0.010134	C8
HMDB0042548	2	TG 46:4	0.11825	-3.0801	0.012234	C8

HMDB0006731	1	CE 20:5	1.5254	0.60923	0.012234	C8
HMDB0005447	2	TG 54:7	0.60078	-0.7351	0.01673	C8
HMDB0010517	2	TG 52:7	0.44856	-1.1566	0.019888	C8
HMDB0042811	2	TG 48:4	0.37734	-1.406	0.023132	C8
HMDB0005380	2	TG 52:5	0.52181	-0.93841	0.023269	C8
HMDB0008038	2	PC 36:1	1.702	0.76725	0.027221	C8
HMDB0009060	2	PE 36:3	0.55382	-0.8525	0.027246	C8
HMDB0005392	2	TG 56:8	0.62665	-0.67428	0.028054	C8
HMDB0007103	2	DG 34:2	0.33022	-1.5985	0.031328	C8
HMDB0010498	2	TG 54:9	0.43841	-1.1896	0.031328	C8
HMDB0042751	2	TG 46:3	0.36555	-1.4519	0.033277	C8
HMDB0005436	2	TG 52:6	0.53192	-0.91072	0.033277	C8
HMDB0002815	2	LPC 18:1	1.595	0.67354	0.036591	C8
HMDB0012097	1	SM 18:1;O2/14:0	1.5153	0.59964	0.042106	C8
HMDB0010471	2	TG 50:5	0.50908	-0.97404	0.04255	C8
HMDB0007098	2	DG 32:0	0.47406	-1.0768	0.048165	C8
HMDB0005410	2	TG 56:3	2.0185	1.0133	0.048165	C8
HMDB0007132	2	DG 34:3	0.18632	-2.4242	0.062545	C8
HMDB0042104	2	TG 51:1	0.49863	-1.004	0.067745	C8
HMDB0010513	2	TG 56:10	0.49326	-1.0196	0.078346	C8
HMDB0011706	2	TG 49:2	0.18173	-2.4601	0.078417	C8
HMDB0010518	2	TG 54:8	0.64366	-0.63562	0.078417	C8
HMDB0005405	2	TG 54:3	1.5814	0.66119	0.081846	C8
HMDB0005403	2	TG 54:2	1.7343	0.79431	0.082694	C8
HMDB0005476	2	TG 58:10	0.60209	-0.73195	0.082694	C8
HMDB0005448	2	TG 56:9	0.66208	-0.59493	0.082694	C8
HMDB0006729	2	CE 22:4	1.6614	0.73241	0.098893	C8
